# Cytotoxic activity in cutaneous leishmaniasis

**DOI:** 10.1590/0074-02760170109

**Published:** 2017-11

**Authors:** Taís M Campos, Rúbia Costa, Sara Passos, Lucas P Carvalho

**Affiliations:** 1 Universidade Federal da Bahia, Serviço de Imunologia, Salvador, BA, Brasil; 2 Universidade Federal da Bahia, Faculdade de Medicina da Bahia, Programa de Pós-Graduação em Ciências da Saúde, Salvador, BA, Brasil; 3 Houston Methodist Research Institute, Department of Nanomedicine, Houston, TX, United States; 4 Instituto Nacional de Ciências e Tecnologia-Doenças Tropicais, Salvador, BA, Brasil; 5 Fundação Oswaldo Cruz-Fiocruz, Instituto Gonçalo Moniz, Laboratório Avançado de Saúde Pública, Salvador, BA, Brasil

**Keywords:** cytotoxicity, CD8+ T cells, NK cells, cutaneous leishmaniasis, immunopathology

## Abstract

Cutaneous leishmaniasis (CL) is a chronic disease caused by species of the protozoan *Leishmania* and characterised by the presence of ulcerated skin lesions. Both parasite and host factors affect the clinical presentation of the disease. The development of skin ulcers in CL is associated with an inflammatory response mediated by cells that control parasite growth but also contribute to pathogenesis. CD8+ T cells contribute to deleterious inflammatory responses in patients with CL through cytotoxic mechanisms. In addition, natural killer cells also limit *Leishmania* infections by production of interferon-γ and cytotoxicity. In this review, we focus on studies of cytotoxicity in CL and its contribution to the pathogenesis of this disease.

The outcome of cutaneous leishmaniasis (CL) depends on the infecting parasite species and the type and intensity of the immune response to that parasite (Barral Netto et al. 1998, Castellano et al. 2009, Kaye & Scott 2011). The coordinated action of innate and adaptive immune responses is fundamental for protection against *Leishmania* (Gorak et al. 1998). As cells in the innate response, natural killer (NK) cells represent an important line of defence against these parasites, acting as the main source of interferon (IFN)-γ early in infection and thus contributing to the activation of macrophages to kill *Leishmania* (Gorak et al. 1998). Susceptibility or resistance to disease is associated with responses mediated by T lymphocytes (CD4+ and CD8+) (Scott et al. 1988) that produce cytokines such as tumour necrosis factor (TNF) and INF-γ, which are important to controlling *Leishmania* infections (Pirmez et al. 1993, Da-Cruz et al. 2002). Moreover, these cells mediate effector mechanisms to fight infections, not only through the secretion of cytokines and chemokines but also through cytotoxic activity that induces apoptosis of infected cells (Ruiz & Becker 2007). Conversely, some studies have shown the contribution of cytotoxic activity to skin ulcer development in patients with CL. Here, we discuss recent literature defining the contribution of cytotoxic cells to the pathogenesis of CL.

## Cytotoxic activity and pathogenesis of CL

CL is caused by several different species of *Leishmania* protozoan parasites, and both parasite and host factors affect the clinical spectrum of the disease (Barral Netto et al. 1998, Castellano et al. 2009, Schriefer et al. 2009). At an early stage of infection, neutrophils are recruited rapidly to the site of inoculation, where they capture *Leishmania* and produce reactive oxygen species and elastase (Peters et al. 2008, Thalhofer et al. 2011, Falcão et al. 2015). Neutrophils also release cytokines such as TNF and chemokines and interact with mononuclear phagocytes, contributing to both resistance and susceptibility to *Leishmania* infections (Scapini et al. 2000, Ribeiro-Gomes et al. 2004, 2007, 2012, Novais et al. 2009). In addition to neutrophils, mononuclear phagocytes interact with *Leishmania* early in infection, playing a pivotal role in promoting resistance to the parasite, mainly through the production of IL-12 that leads to differentiation of naive CD4+ T cells into Th1-type T cells. IL-12 is also important in inducing IFN-γ secretion by NK cells, which can promote the destruction of *Leishmania* through a cytotoxic mechanism (Gorak et al. 1998, Lemos et al. 2004). Although *Leishmania* parasites reside within parasitophorous vacuoles in mononuclear phagocytes, their antigens are presented via MHC class I to CD8+ T cells, which contributes to a Th1 environment through the production of TNF and IFN-γ (Pirmez et al. 1993, Da-Cruz et al. 2002, D'Oliveira Jr et al. 2002, Jordan & Hunter 2010). Moreover, cytotoxic activity by CD8+ T cells is important to parasite elimination (Khan et al. 1990, Da-Cruz et al. 1994, Barral Netto et al. 1995, Montoya et al. 1996, Purner et al. 1996, Jordan & Hunter 2010). While cytotoxic activity induces target cell death, cytokines such as IFN-γ and TNF participate in the development of an inflammatory response that modulates macrophage and dendritic cell activity; however, when these pathways are not properly regulated, inflammatory disorders and tissue damage can develop (Ribeiro-de-Jesus et al. 1998, Follador et al. 2002, Arias et al. 2014). This is the scenario observed in patients with CL: after *Leishmania* infection, most patients develop lymphadenopathy, followed by the appearance of a papule at the bite site (Barral et al. 1992, 1995), with higher parasite load (Saldanha et al. 2017). In a few days, the papule becomes an ulcerated lesion characterised by intense inflammatory infiltration, with presence of T and B lymphocytes, mononuclear phagocytes, plasma cells (Pirmez et al. 1993, Da-Cruz et al. 2002), and a few parasites (Ribeiro-de-Jesus et al. 1998, Bacellar et al. 2002, Gaze et al. 2006, Saldanha et al. 2017). High levels of the proinflammatory cytokine TNF has been detected in the biopsies of patients with CL (Antonelli et al. 2005). This cytokine contributes to the destruction of *Leishmania* but also induces cellular adhesion, necrosis, and cytotoxicity, thereby contributing to disease pathogenesis (Ribeiro-de-Jesus et al. 1998, Bacellar et al. 2002, Da-Cruz et al. 2002). In this sense, CD8+ T cells and NK cells participate in the immune response to *Leishmania braziliensis,* not only contributing to TNF production but also showing cytotoxic activity. Machado et al. (2002) observed CD8+ T cells and NK cells and their cytotoxic activities in lesions of patients with CL, suggesting not only the active participation of these cells in parasite destruction but also their role in ulceration. IL-10 is the main regulatory cytokine in this immune response, and despite of the presence of IL-10 in ulcers of CL and mucosal leishmaniasis (Tomlinson et al. 2008) cases, low levels of expression of its receptor have been documented and associated with a high frequency of activated CD4+ and CD8+ T cells (Bacellar et al. 2002, Faria et al. 2005, Gaze et al. 2006, Carvalho et al. 2007).

Recent work has shown that cytotoxicity is one of the main mechanisms underlying disease induced by *L. braziliensis* infection (Faria et al. 2009, Dantas et al. 2013, Novais et al. 2013, 2015, Santos et al. 2013, 2015, Cardoso et al. 2015, Ferraz et al. 2015, 2017) (Table). In a recent study performed by our group, we compared gene expression in lesions of patients with CL with that in normal skin from healthy individuals. Genes associated with cytolysis, such granzyme B, granzyme A, and granulysin, were highly expressed in CL lesions when compared to normal skin. This study revealed a significant enrichment in the activity of pathways involved in NK cell-mediated cytotoxicity in CL lesions (Novais et al. 2013). Interestingly, we also found that NK cell cytolytic pathways activated in CL lesions were greatly enriched when compared to those in skin lesions of patients with psoriasis, suggesting that cytolysis is a pathological characteristic associated with skin ulcers but not skin inflammatory plaques. Another transcriptional analysis of lesions showed that early non-ulcerated papular lesions from CL individuals had a similar transcriptional profile to that of ulcerated lesions, indicating that the pathological response occurs very soon after infection in patients with CL (Novais et al. 2015). These results suggest that cytotoxicity is one of the main mechanisms of immunopathology in CL, rather than being a consequence of pathology.

**TABLE:** Contribution of cytotoxic activity for pathogenesis of human cutaneous leishmaniasis

Authors	Specimen type	Infection	Clinical dorm	Granzyme/Perforin/CD107a	Main results
Faria et al. (2009)	Lesion cells	*Leishmania braziliensis*	CL	Granzyme A	Expression of granzyme A by CD8^+^ T cells is involved in tissue destruction.
Santos et al. (2013)	*Ex-vivo/In-vitro:* blood lesion cells	*L. braziliensis*	CL	Granzyme B CD107a	Frequency of granzyme B^+^ correlates positively with lesion size; increased CD107a expression on CD8^+^ T cells upon infection.
Dantas et al. (2013)	*In situ:* lesion cells	*L. braziliensis*	ECL, CL and DL	Granzyme B	ECL: positive correlation between granzyme B^+^ cells and inflammatory infiltrate in lesions.
Novais et al. (2013)	*Ex-vivo* lesion cells	*L. braziliensis*	CL	Granzyme B Perforin CD107a	CD8^+^ T cells from lesions express more granzyme B, perforin and CD107a than CD8^+^ T cells from peripheral blood.
Cardoso et al. (2015)	*Ex-vivo/In-vitro:* blood	*L. braziliensis*	CL SC	Granzyme B	Granzyme B production in CD8^+^ T cells is higher in CL than in SC individuals.
Novais et al. (2015)	Lesion cells	*L. braziliensis*	CL	Granzyme B Perforin	Transcripts associated the cytotoxicity and apoptosis (GZMB, PRF1, CASP3, CASP4, CASP5, CASP7 and BID).
Ferraz et al. (2015)	*Ex-vivo/In-vitro:* blood	*L. braziliensis*	CL-during treatment		Positive correlation between frequency of apoptotic-effector CD8^+^ T cells and lesion size.
Ferraz et al. (2017)	*Ex-vivo:* blood	*L. braziliensis*	CL	CD107a	NKT and double negative T cells are the main cell population of degranulating in CL lesions.

CL: cutaneous leishmaniasis; DL: disseminated leishmaniasis; ECL: early cutaneous leishmaniasis; SC: subclinical infection.

Cytotoxic activity is shown by a variety of immune cells, including CD8+ T, NK, and NKT cells, that destroy targets through the release of cytotoxic granules containing preforin and granzymes. Perforin promotes pore formation in target cell membranes, facilitating the entry of granzymes and inducing programmed cell death through DNA fragmentation (Trapani 2001, Trapani & Smyth 2002, Pipkin & Lieberman 2007, Ruiz & Becker 2007). Cell death can occur through the activation of apoptotic cysteine proteases (caspases) or in the absence of their activation (Waterhouse et al. 2006, Pipkin & Lieberman 2007, Hoves et al. 2010). Activation of the mitochondrial pathway by granzyme B-inducing cell death mechanisms has been observed in CL lesions, and a positive correlation between the expression of caspase-3 and caspase-9, as well as caspase-9 and granzyme B, was observed in individuals with these lesions (Santos et al. 2015). The caspase pathway is also associated with lesion progression, with a positive correlation between the expression of caspase-3, caspase-9, and granzyme B proteins and lesion size in patients with CL (Santos et al. 2015). Although the effect of cytotoxic mechanisms is the destruction of target cells, CD8+ T cell cytotoxicity does not control *L. braziliensis* parasites (Santos et al. 2013). Santos et al. (2013) found that CD8+ T cells in co-culture with Leishmania-infected macrophages released granzyme B but had no effect on parasite killing, whereas CD4+ T cells in co-culture with infected macrophages produced IFN-γ and mediated *Leishmania* killing. Additionally, an association between lesion size and the presence of cytotoxic cells has been documented in *L. braziliensis* infections. Faria et al. (2009) reported that the frequency of CD8+ T cells expressing granzyme is directly associated with the intensity of inflammation in ulcers of patients with CL. Furthermore, patients with ulcerated CL lesions have a higher frequency of these cells than patients in the initial phase of infection, indicating the participation of CD8+ T cells in disease progression. A histopathological analysis of fragments of lesions from patients with early CL, late CL, and disseminated leishmaniasis (DL) showed the presence of CD8+ granzyme B+ lymphocytes in the papillary dermis. Further, an evaluation of cytotoxic activity in inflammatory infiltrates revealed that new lesions (those with less than 20 days of development) had fewer cells expressing granzyme B than late ulcers and ulcers from patients with DL (Dantas et al. 2013). Taken together, these data suggest that cytotoxic activity of CD8+ T cells and granzyme B production can lead to injury of the basal membrane layer, contributing to ulcer formation and disease progression.

Some individuals residing in areas with *L. braziliensis* transmission do not have a history of leishmaniasis but are *Leishmania* skin test-positive but have no symptoms (Follador et al. 2002). The ratio of infection to disease in one CL endemic area in northeastern Brazil is 3.7:1 (Unger et al. 2009). An evaluation of the cytotoxic activity of CD8+ T cells in sub-clinical infections and patients with CL showed that CD8+ T cells in individuals with CL induced apoptosis of more infected monocytes than CD8+ T cells from sub-clinically infected subjects, and the production of granzyme B in CD8+ T cells was higher in individuals with CL than in those with sub-clinical infection (Cardoso et al. 2015). This suggests that cytotoxic activity of CD8+ T cells in patients with CL can contribute to pathology, because subjects with sub-clinical infections do not develop lesions.

To date, the mechanisms underlying cytotoxicity-induced tissue damage in CL are not well understood; however, a few studies have demonstrated the participation of cytolytic molecules in deleterious immune responses to *L. braziliensis* infection. Lytic granules of cytotoxic lymphocytes are composed of secretory lysosomes with a dense centre of granzyme and perforin proteins (Trapani & Smyth 2002, Thiery et al. 2011). This centre is covered with a lipid bilayer composed of lysosomal-associated membrane protein 1 (LAMP-1), also known as CD107a (Betts et al. 2003). Tissue from CL lesions cultured with *L. braziliensis* parasites showed an increase in CD107a expression relative to that of non-infected control tissue from the same lesion (Santos et al. 2013). Furthermore, this study demonstrated the localisation of CD8+ T cells and granzyme B in biopsied tissue from patients and a positive correlation between the amounts of CD8+ T cell granzyme B+ and lesion size (Santos et al. 2013). We also showed that CD8+ T cells from CL lesions express CD107a, whereas CD8+ T cells from the blood of patients with CL do not express this protein (Novais et al. 2013). Another mechanism underlying cytotoxicity that does not depend on MHC class I antigen presentation is the engagement of NKG2D, which is present in a variety of cells, including CD8+ T cells and NK/NKT cells.

Although several cell types are cytotoxic, few studies have shown the contribution of cells other than CD8+ T cells in cytolysis in *Leishmania.* Cytotoxic activity of NK cells is initiated after recognition of ligands present on target cells by the receptors NKp30, NKp44, NKp46, and NKG2D, with the latter expressed by CD8+ T cells (Watzl 2003, Lanier 2005). Studies on Nk cells have shown that the most abundant surface glycoprotein on *Leishmania,* gp63, inhibits human NK cell proliferation and decreases expression of NKp30, NKp44, and CD16 (Lieke et al. 2008). In contrast, *Leishmania* lipophosphoglycan (LPG) activates NK cells via Toll-like receptor 2, leading to the production of the proinflammatory cytokines IFN-γ and TNF (Becker et al. 2003). The recognition of LPG by NK cells was also shown to lead to promastigote lysis, but to destroy NK cells in a non-apoptotic manner (Lieke et al. 2011). Recently, Naouar et al. (2014) evaluated the cytotoxic immune responses of cells from individuals with previous contact with *Leishmania major.* They observed that CD4+ T cell subsets produced granzyme B in response to *L. major* antigens. Although this result suggests a cytotoxic role for CD4+ T cells, cytolysis was not assessed in these experiments. Previous studies have shown that the main role of CD4+ T cells in *L. braziliensis* infections is IFN-γ production for parasite killing (Kaye & Scott 2011, Santos et al. 2013). Ferraz et al. (2017) evaluated the frequency of degranulating cell subsets in CL lesions and found that NKT cells and double-negative T cells (CD3+CD4-CD8-) displayed a higher frequency of CD107a than did CD8+ T cells.

Mechanisms other than cell death by apoptosis, such as excessive degradation of the extracellular matrix (ECM), may also indirectly contribute to leishmaniasis pathology. (Costa et al. 2008, Maretti-Mira et al. 2011a, b, Campos et al. 2014). Granzymes have been shown to activate pro-inflammatory cytokines and degrade multiple components of the ECM (Hiebert & Granville 2012). Treatment of epithelial cells, fibroblasts, or monocytes with purified human granzyme A resulted in the production of pro-inflammatory cytokines such as IL-1β, IL-6, IL-8, and TNF in a process that may be dependent on caspase-1 (Sower et al. 1996, Metkar et al. 2008). Granzyme B can indirectly promote inflammation through the activation of cytokines such as IL-18, IL-1β, and IL-1α (Omoto et al. 2010, Afonina et al. 2011, Hiebert & Granville 2012). One likely explanation of how cytotoxic cells mediate inflammation and tissue injury in CL is that, after degranulation of cytotoxic cells, granzyme B and perforin are released into the extracellular space, inducing apoptosis of infected macrophages and bystander cells. Additionally, extracellular granzyme B may indirectly induce inflammation through the activation of pro-inflammatory cytokines and degradation of ECM substrates, contributing to tissue injury (Figure). These data suggest a role for granzymes in amplifying inflammation in *Leishmania* infections and thus contributing to tissue damage. However, additional functional studies must be performed to elucidate the role of granzymes in human cases of CL.

**Figure:**
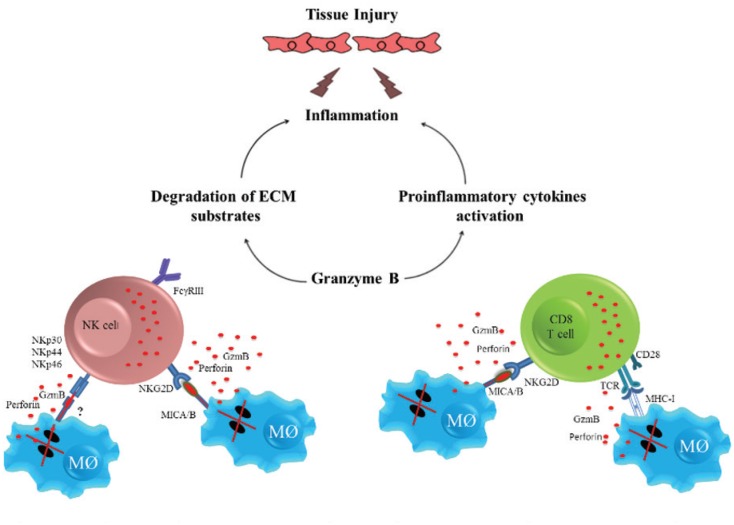
Tissue damage and inflammation mediated by cytotoxic cells in human cutaneous leishmaniasis. With degranulation of natural killer (NK) and CD8+ T cells, granzyme B (GzmB) and perforin are released and apoptosis of infected macrophages is induced. Additionally, granzyme B may indirectly induce inflammation through the activation of pro-inflammatory cytokines and degradation of extracellular matrix (ECM) substrates, contributing to tissue damage. *This image has not been previously published.

## Cytotoxic activity in experimental models of CL

Mouse models have been used to study many aspects of innate and adaptive immune responses to various *Leishmania* species. Initially, CD8+ T cells were associated with protection and cure of leishmaniasis, as studies using experimental models of leishmaniasis showed that CD8+ cells were required for a primary immune response against *L. major* infection (Titus et al. 1987, Hill et al. 1989, Muller et al. 1991). Later, these data were contradicted, when mice deficient in CD8 or β2-microglobulin were shown capable of resolving primary *L. major* infection (Overath & Harbecke 1993, Wang et al. 1993, Huber et al. 1998). A few years later, a protective response induced by CD8+ T cells was again suggested in models of infection with a low dose of *L. major.* In these models, IFN-γ produced by CD8+ cells was essential for controlling primary infection (Belkaid et al. 2002, Uzonna et al. 2004).

More recently, however, a deleterious effect of CD8+ T cells, mainly as a result of its cytotoxic activity, has been observed. Experiments using mouse models have contributed to a better understanding of cytotoxicity-induced pathologic responses in CL. Novais et al. (2013) showed the deleterious effect of CD8+ T cells on *L. braziliensis*-infected mice, in which disease progression and metastasis were associated with the presence of CD8+ T cells and perforin, indicating that the cytotoxic activity of CD8+ T cells promotes pathology rather than being a consequence of the disease. The same group showed that memory bystander CD8+ T cells expressing NKG2D and that were not specific to *Leishmania* antigens were able to infiltrate lesions and contribute to immunopathology in mice infected with *L. major* (Crosby et al. 2014). In another study, mice previously infected with viral or bacterial pathogens and then infected with *L. major* developed significantly larger lesions with an increased number of NKG2D+ CD8+ T cells. In this case, depletion of CD8+ T cells, as well as the blockage of NKG2D, decreased the size and severity of lesions, suggesting that the immunopathology observed in LCMV/L. *major* co-infected mice was dependent on cytotoxic CD8+ T cells induced by NKG2D ligation. In the same study, lesions with a small number CD8+ T cells produced IFN-γ, whereas high number these cells expressed granzyme B (Crosby et al. 2015). These data indicate that the mechanisms underlying cell death induced by cytotoxic cells contribute to tissue damage instead of parasite killing. In addition, a study by our group indicated that genes associated with cytotoxicity and inflammasome activation were up-regulated in lesions from humans infected with *L. braziliensis* (Novais et al. 2015). Interestingly, IL-1β production in these lesions was dependent on CD8+ T cell cytotoxicity (Novais et al. 2017). These data support an association between cytotoxic activity and inflammation in cutaneous leishmaniasis. The controversy between a protective versus pathologic role for CD8+ T cells in cutaneous leishmaniasis clearly depends on the parasite dose, as well as the site where the immune response was assessed. Earlier studies investigated the contribution of lymph node and spleen CD8+ T cell to protection, whereas later studies assessing immune response in lesions revealed a deleterious role for CD8+ T cells. The different roles for CD8+ T cells in leishmaniasis may reflect heterogeneity in the CD8+ T cells population; however, this hypothesis requires further investigation.

The role of NK cells in protection against many pathogens has been a focus of study because of their ability to rapidly produce cytokines and lyse target cells without prior sensitisation. In mouse CL, NK cells have been associated with protection, mainly through the production of IFN-γ, which is important for macrophage activation (Laskay et al. 1993, Laurenti et al. 1999, Muller et al. 2001). Furthermore, cytotoxic activity and IFN-γ production by NK cells was also observed in lymph nodes of self-healing *L. major*-infected mice (Scharton & Scott 1993, Bajenoff et al. 2006, Liese et al. 2007). In contrast, NK cells were not necessary for the development of protective immunity against *L. major,* as, in mice lacking NK cells (NK-T+), an efficient Th1 response with high production of IL-12 and IFN-γ and control of parasites was observed (Satoskar et al. 1999). In addition, in mice lacking CD4+ T cells, IFN-γ production by NK cells was not sufficient to control *L. major* (Wakil et al. 1998). Although these studies are controversial regarding the requirement for NK cells to control infection, the fact that these cells are potent producers of IFN-γ suggest their protective role in cases of CL.

Despite evidence suggesting cytotoxicity as an immune mechanism that contributes to CL pathogenesis, the factors associated with severity of disease still need to be determined.

## Concluding remarks

Taken together, the literature shows that NK cells and T lymphocytes not only participate in the control of *Leishmania* proliferation through IFN-γ production but also that these cells may be involved in skin ulceration through tissue disruption as a result of their cytotoxic activity.
